# Liquid-liquid phase separation regulates alpha-synuclein aggregate and mitophagy in Parkinson’s disease

**DOI:** 10.3389/fnins.2023.1250532

**Published:** 2023-09-14

**Authors:** Kaiying Hou, Tingting Liu, Jingwen Li, Meiyan Xian, Lin Sun, Jianshe Wei

**Affiliations:** ^1^School of Life Sciences, Henan University, Kaifeng, China; ^2^College of Chemistry and Molecular Sciences, Henan University, Kaifeng, China

**Keywords:** liquid-liquid phase separation, Parkinson’s disease, mitophagy, alpha-synuclein, PINK1-Parkin

## Abstract

Parkinson’s disease (PD) is the second most common neurodegenerative disease in the world, and alpha-synuclein (α-syn) abnormal aggregate and mitochondrial dysfunction play a crucial role in its pathological development. Recent studies have revealed that proteins can form condensates through liquid–liquid phase separation (LLPS), and LLPS has been found to be widely present in α-syn aberrant aggregate and mitophagy-related protein physiological processes. This review summarizes the occurrence of α-syn LLPS and its influencing factors, introduces the production and transformation of the related protein LLPS during PINK1-Parkin-mediated mitophagy, hoping to provide new ideas and methods for the study of PD pathology.

## 1. Introduction

### 1.1. Parkinson’s disease

Parkinson’s disease (PD) is the second most common neurodegenerative disease in the world, with approximately 6.1 million people affected worldwide in 2016. PD occurs mainly in older age groups, with a prevalence of about 0.5–1% in people aged 65–69 years, rising to 1–3% in people aged 80 years and older, and with a higher prevalence in men than in women (Tanner and Goldman, 1996; GBD 2016 Parkinson’s Disease Collaborators, 2018). With the aging of the population, the prevalence and incidence of PD are projected to increase by 30% by 2030, posing a heavy burden on social development (Chen et al., 2001).

Patients with PD often exhibit motor and non-motor symptoms. Motor symptoms such as bradykinesia, rigidity, resting tremor, postural instability; and non-motor symptoms include loss of smell, sleep disturbance, autonomic dysfunction, psychological disorders, cognitive impairment, etc. (Kouli et al., 2018; Armstrong and Okun, 2020). The exact cause of PD is still unknown, and studies have shown that its occurrence may be related to various factors such as genetics, environment, and lifestyle. The main pathological features of PD are abnormal aggregate of alpha-synuclein (α-syn) to form Lewy bodies and progressive loss of dopaminergic neurons in the substantia nigra compacta. Mitochondrial dysfunction is also thought to play a vital role in the development of PD pathology. Mitochondria produce ATP through the process of oxidative phosphorylation, which is the primary source of intracellular energy production, and impaired mitochondrial function in PD patients leads to a reduction in energy production, which negatively affects neuronal function and survival. In addition, mitochondria are the main source of oxidative stress, and the generation of reactive oxygen radicals can cause damage to cellular components (such as proteins, lipids, and DNA), and mitochondrial dysfunction in PD exacerbates oxidative stress, further triggering cytotoxic and inflammatory responses (Grunewald et al., 2019; Monzio Compagnoni et al., 2020; Malpartida et al., 2021). When mitochondria are damaged, cells degrade the damaged mitochondria through selective autophagy (i.e., mitophagy) to regulate cellular homeostasis.

There is a close relationship between α-syn and mitophagy, which plays an essential role in the pathogenesis of PD. Mitophagy can remove abnormally aggregated α-syn, and enhanced mitophagy reduces α-syn aggregate, thereby attenuating the pathological progression of PD (Picca et al., 2021). Moreover, the abnormal aggregate of α-syn can interfere with the normal function of mitophagy-associated proteins (e.g., PINK1, Parkin), thus affecting the mitophagy process (Minami et al., 2015).

Currently, PD treatment can only relieve patients’ symptoms rather than cure them. Therefore, exploring the cause of the disease and clarifying the pathological process are crucial for furthering understand PD and finding appropriate treatments. Further exploration of the roles played by α-syn and mitophagy in the pathology of PD, and their interactions, has become an important starting point to clarify the pathogenesis of PD.

### 1.2. Liquid–liquid phase separation (LLPS)

Recent studies have revealed a close relationship between phase separation and PD. Such as oil drops in water, the process by which different components of a liquid environment are separated to form two or more distinct phases under certain conditions due to differences in their biophysical properties is known as liquid–liquid phase separation (LLPS). In living cells, biomolecules (proteins or nucleic acids) are separated by LLPS into liquid-like, non-membranous bodies (called phases, also known as biomolecular condensate) with specific functions, and there are distinct interfaces between the different phases to form separate compartments isolated from the external environment, ensuring that different biochemical reactions take place in time- and space-constrained compartments (Wang and Zhang, 2019; Gouveia et al., 2022). An increasing number of studies have shown that LLPS is involved in the formation of intracellular membraneless organelles, such as P granules (Brangwynne et al., 2009), nucleolus (Brangwynne et al., 2011), stress granule proteins (SG) (Molliex et al., 2015) etc. LLPS of biomacromolecules (proteins or nucleic acids) has become an important mechanism to assist cellular functions. In a solution containing a biomolecule, biomolecules and solvent molecules tend to be evenly distributed to maintain the maximum entropy value and the lowest free energy (Alberti et al., 2019). When the concentration of biomacromolecules gradually increases, the biomacromolecules gradually aggregate and self-assemble to form a concentrated phase due to multivalent interactions between molecules, especially weak interactions. In this case, their reduced entropy value is compensated by the additional molecular interactions in the concentrated and diluted phases (Zhang et al., 2020). The possession of intrinsically disordered regions (IDR) and low-complexity domains (LCD) is one of the characteristics of proteins capable of LLPS, with IDR lacking a stable conformation that contributes to the involvement of intermolecular interactions. LCD is characterized by amino acid bias and/or repetitive linear motifs, a feature often associated with structural disorders of proteins (Molliex et al., 2015).

Biomolecules form condensate through LLPS, the condensate is not static after its formation and will change as the external environment changes and the internal structure is adjusted (Wang and Zhang, 2019). In the initial stage of LLPS to form a condensate, the mutual gravitational force between the liquid molecules is not sufficient to hold the entire liquid phase clumps together, and these condensates float in solution in the form of a liquid with a high degree of mobility. With the passage of time or under the action of various influencing factors, the intermolecular gravitational force inside the condensate gradually increases, and the structure of the droplet gradually becomes more organized. This ordered structural state is called gel-like, gel-like has a certain degree of elasticity and solid nature, but still has a certain degree of mobility. Based on gel-like formation, further interactions can lead to re-aggregate of the gel to form fibrous aggregate (Alberti et al., 2019). The size, formation rate, and biophysical properties of phase-separated protein condensate are important for their realization of different cellular functions, and such properties change when influenced by different factors, such as the transition from a highly fluid liquid state to a hydrogel state and eventually to a solid-like condensate (Banani et al., 2017; Alberti et al., 2019). In addition, multiple factors (e.g., environmental changes, changes in the protein itself, protein–protein interactions, etc.) have been found to affect the physiological functions of proteins/RNA by influencing the LLPS process of biomolecules (Ray et al., 2020; Poudyal et al., 2022; Huang et al., 2022a).

In addition, LLPS also plays a vital role in the study of human pluripotent stem cells (hPSCs), where many key transcription factors and RNA-binding proteins can undergo phase separation phenomena to form condensate that influences the expression of specific genes and cell fate decisions and is involved in the regulation of the process that maintains the stemness and self-renewal capacity of hPSCs (Kim, 2021; Lim and Meshorer, 2021).

### 1.3. Relationship between LLPS and neurodegenerative diseases, and PD

Recently, we found that multiple neurodegenerative disease-associated proteins undergo the biophysical process of LLPS, such as Tau protein in Alzheimer’s disease (AD) (Ambadipudi et al., 2017; Wegmann et al., 2018; Kanaan et al., 2020; Wen et al., 2021), α-syn in PD (Hardenberg et al., 2020; Ray et al., 2020), FUS (Patel et al., 2015; Zbinden et al., 2020) and TDP-43 (Conicella et al., 2016; Liu and Fang, 2019) associated with amyotrophic lateral sclerosis (ALS) and frontotemporal dementia (FTD), etc. The current focus is mainly on α-syn and phase separation processes in mitophagy. LLPS is observed to occur in the early stages of α-syn aberrant aggregate (Elbaum-Garfinkle, 2019; Ray et al., 2020), the formation of biomolecular condensates by α-syn phase transition is closely related to the pathogenesis of PD. In addition, LLPS has been found to be involved in the regulation of mitophagy processes (Noda et al., 2020; Yamasaki et al., 2020; Peng P. H. et al., 2021; Xing et al., 2021; Brodin et al., 2022). LLPS in α-syn and mitophagy are discussed further below. Therefore, it is important to observe and study the role of LLPS in α-syn abnormal aggregate in PD and its role in regulating mitophagy for our further understanding of the pathogenesis of PD.

This review generalizes the occurrence, development, and related influencing factors of α-syn LLPS, a key pathological protein in PD, and the research progress of LLPS in PINK1-Parkin-mediated mitophagy, starting from the mechanism of the role of biomolecular condensates in the process of α-syn aggregate and mitophagy in PD, and discusses its importance in the development of PD pathology, hoping to provide new ideas and methods for the pathological research of PD.

## 2. LLPS of the pathological protein α-syn in PD

### 2.1. Occurrence of α-syn LLPS

α-syn misfolding and aggregate is a key target for PD treatment. Therefore, observing the role of α-syn LLPS in aggregate is crucial for us to clarify the pathogenesis of PD further (Giampa et al., 2021; Gadhe et al., 2022; Li et al., 2022). α-syn is a naturally unfolded protein consisting of 140 amino acids, which is abundantly present in presynaptic nerve endings, and consists of three structural domains: a positively charged amphiphilic N-terminal domain (residues 1–60), which interacts with the membrane; a hydrophobic non-amyloid beta component (NAC) domain (residues 61–95), which is involved in fiber formation and aggregate; and a negatively charged acidic C-terminal domain (residues 96–140), associated with α-syn nuclear localization and involved in the interaction of α-syn with metal ions, ligands and other proteins (Uversky and Eliezer, 2009; Poudyal et al., 2022). It was found that the LCD in the N-terminal and NAC domains of α-syn are the key factors driving the phase separation of α-syn, but exactly which residues are involved is still unknown.

*In vitro*, expression of purified α-syn undergoes LLPS and occurs before α-syn aggregate. The formation of liquid-like condensates of α-syn in the presence of 10% polyethylene glycol (PEG)-8,000 at a concentration of ≥200 μm was observed by differential interference contrast microscope (DIC), which was further confirmed by light scattering and fluorescence imaging using fluorescein isothiocyanate (FITC)-labeled α-Syn (10% labeled). Over time, α-syn condensates undergo an abnormal phase transition from liquid (day 2) to gel (day 5) to solid-state (day 30), with reduced mobility and migration capacity, eventually giving rise to amyloid fibril aggregate. Also, α-syn condensates were observed to appear in the cellular model and transform into perinuclear aggregates (Ray et al., 2020). This phenomenon was also observed in nematodes, when yellow fluorescent protein (YFP)-tagged human α-syn protein was stably expressed in nematode body wall muscle cells, the emergence of condensates in nematode adults was observed by high-resolution fluorescence lifetime imaging microscopy (FLIM), and the liquid state (days 1–11) was gradually transformed into a starch-rich hydrogel with the nature of Louisianian vesicles as nematodes grew (days 13–15) (Hardenberg et al., 2021).

### 2.2. Multiple factors affect the α-syn LLPS process

#### 2.2.1. Experimental conditions affect LLPS

**Figure 1:**
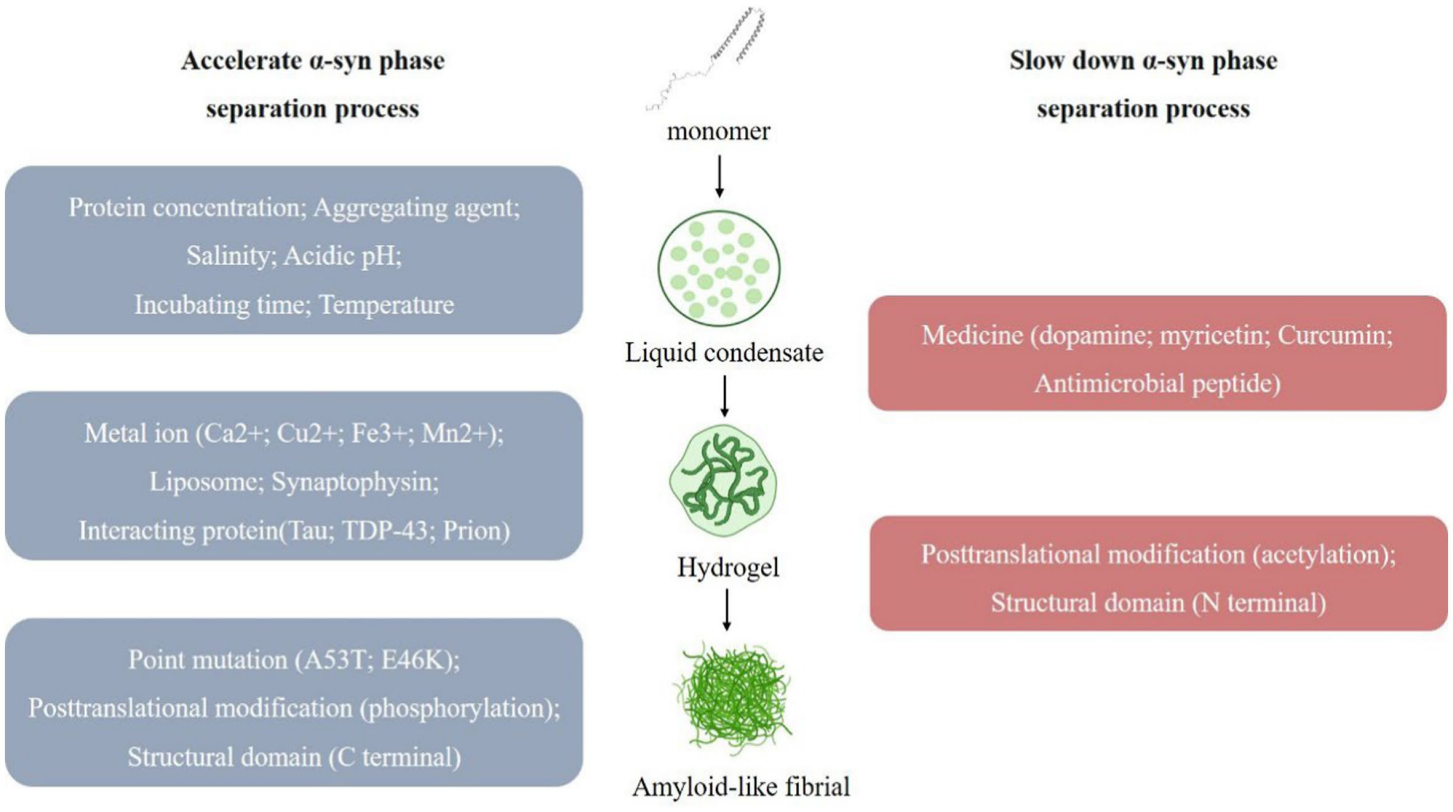
Multiple factors affect the α-syn Liquid-liquid phase separation (LLPS) process. α-syn undergoes LLPS to form liquid condensates, and under the influence of various factors, the state changes, forming hydrogel and eventually forming amyloid-like fibril. (1) Multiple factors promote α-syn LLPS. (i) Experimental conditions, such as protein concentration; aggregating agent; salinity; acidic pH; incubating time; temperature. (ii) External substances, such as metal ion (Ca2+; Cu2+; Fe3+; Mn2+); liposome; synaptophysin; interacting protein (Tau; TDP-43; Prion). (iii) α-syn own change, such as point mutation (A53T; E46K); posttranslational modification (phosphorylation); structural domain (C terminal). (2) Multiple factors slow down α-syn LLPS. (i) External substances, such as medicine (dopamine; myricetin; curcumin; antimicrobial peptide). (ii) α-syn own change, such as posttranslational modification (acetylation); structural domain (N terminal).

Changes in the reaction system such as protein concentration, aggregates, salt concentration of the buffer system, pH, time, temperature, etc. can have certain effects on α-syn LLPS (Figure 1). When observing the process of protein LLPS, a certain amount of molecular aggregates (e.g., PEG8000, etc.) is often added to simulate the intracellular physiological environment, and usually the higher the protein concentration (e.g., α-syn concentration ≥ 200 μm) and molecular aggregates concentration (PEG-8000 ≥ 10%, w/v), the faster LLPS occurs (Ray et al., 2020; Sawner et al., 2021). In addition, the LLPS of α-syn *in vitro* is affected by the salt concentration of the buffer system, when salt is present (≥500 mM NaCl), the N-terminal and C-terminal of protein molecules are neutralized and the hydrophobic effect is enhanced, which contributes to the occurrence of LLPS (Sawner et al., 2021). The higher the ionic strength, the higher the concentration of protein required for LLPS to occur. The critical concentration for LLPS to occur in α-syn under acidic pH (pH = 5.5) conditions is reduced, which may be related to its closer proximity to the isoelectric point of α-syn. The size and physical properties of α-syn condensates also change with increasing time (day 0–day 30) and temperature (4°C–37°C). Experimental utensils were also found to affect the LLPS process. When the slides were treated with Pluronic F-127, the surface hydrophilicity of the slides increased, LLPS was enhanced, and α-syn aggregate increased (Sawner et al., 2021).

In addition, α-syn LLPS has been associated with a variety of factors that may be involved in regulating the onset of PD by affecting α-syn LLPS processes and altering the normal biological function of α-syn, these are described below (Mukherjee et al., 2022).

#### 2.2.2. PD-related factors affect α-syn LLPS

Metal ions affecting PD pathology (Ca2^+^, Cu^2+^, Fe^3+^, Mn2^+^) were found to accelerate the progression of α-syn LLPS, and this promoting effect could be reversed by the corresponding metal ion chelators (Ray et al., 2020; Sawner et al., 2021; Huang et al., 2022b; Xu et al., 2022b). Ca2^+^ interacts with the acidic region of the C-terminus of α-syn to improve the binding of α-syn to the membrane (Tamamizu-Kato et al., 2006), and also makes the structure of the α-syn monomer more open, which promotes intermolecular electrostatic and hydrophobic interactions and thus LLPS (Han et al., 2018).

Adao, R. found that lipids may be associated with α-syn in PD and that negatively charged lipids induce α-syn folding and promote α-syn aggregate (Adao et al., 2020). At the same time, we observed that the α-syn condensates also recruits lipid membranes, and the N-terminal of α-syn binds to negatively charged lipids, resulting in a tighter conformation of α-syn that accelerates α-syn LLPS and the formation of fibrous aggregate (Fusco et al., 2014).

Synaptophysin is related to the membrane fusion and release of synaptic vesicles. The abnormal aggregate of α-syn may interfere with synaptophysin’s normal function and affect synaptic vesicles’ transport and release process, thereby affecting neurotransmission. Synapsins have also been found to affect the α-syn LLPS process, and when synapsin 1 and α-syn are co-expressed in cells, condensates rich in both proteins appear. Synapsin 1 condensates are poorly mobile and are thought to act as scaffolding molecules for this condensate, α-syn condensates are more mobile and are recruited to aggregate within the condensates, and synaptic vesicles (SVs) are involved in the regulation of this process (Hoffmann et al., 2021).

Multiple drugs for PD affect α-syn LLPS, such that α-syn does not undergo LLPS in the presence of dopamine (an inhibitor of α-syn aggregate) (Ray et al., 2020); yohimbine (a polyhydroxyflavonol compound) delays the α-syn liquid–solid phase transition in a dose-dependent manner, which in turn inhibits amyloid aggregate, and also breaks down amyloid fibrils in mature α-syn condensates (Xu et al., 2022c). Curcumin prevented the transformation of α-syn into amyloid by reducing the mobility of α-syn condensate and also delayed the phase separation transition of PD-associated α-syn E46K and H50Q mutants (Xu et al., 2022a). The antimicrobial peptide LL-III interacts with α-syn monomer and condensate bodies to stabilize the condensate state of α-syn and prevent its transition to the fibrillar state (Oliva et al., 2021).

Several proteins have also been observed to be involved in α-syn LLPS. e.g., the proline-rich P2 region of tau protein interacts with the α-syn C-terminus to recruit α-syn into tau condensates (Siegert et al., 2021). TDP-43 prion-like structural domain monomers promote fibril formation and exhibit enhanced cytotoxicity when co-incubated with α-syn (Dhakal et al., 2021; Agarwal et al., 2022). The interaction of the positively charged N-terminal of Prion protein with the negatively charged C-terminal of α-syn synergistically promotes LLPS condensate formation and liquid-to-solid transition (Agarwal et al., 2022).

#### 2.2.3. α-syn own change affects α-syn LLPS

Mutations of α-syn are closely related to the occurrence and development of PD, and the most common ones are A53T and E46K. These mutations will increase the abnormal aggregate of α-syn, leading to synaptic toxicity, neuronal degeneration, and cell death. α-syn point mutations A53T, E46K also contributes to α-syn LLPS and subsequent fiber formation (Ray et al., 2020). In addition, protein post-translational modifications (PTM) are involved in α-syn LLPS regulation. N-terminal acetylation increases protein solubility and delays α-syn LLPS; S129 phosphorylation accelerates α-syn LLPS and the subsequent liquid-to-solid to amyloid transition (Ray et al., 2020). The different structural domains of α-syn are also involved in the LLPS process. Electrostatic interactions at the C-terminus regulate α-syn LLPS, and truncated α-syn accelerates the aggregate of α-syn amyloid through phase separation (Gallardo et al., 2020; Huang et al., 2022a). In addition, the positively charged N-terminal also interacts with the negatively charged C-terminal over long distances, protecting the NAC region and thus self-inhibiting LLPS (Sawner et al., 2021). When α-syn undergoes LLPS, its conformation changes from a “hairpin” structure to an “elongated” structure, which becomes highly flexible and disordered, promoting protein interactions and LLPS (Ubbiali et al., 2022).

### 2.3. Synaptic nuclear protein family members and α-syn LLPS

β-syn, γ -syn is a member of the same synaptic nuclear protein family as α-syn and is also closely associated with the pathogenesis of PD. β-syn is a 134 amino acid protein with extensive synaptic co-localization with α-syn. It cannot aggregate alone due to the lack of hydrophobic residues 73–83 in its NAC region. β-syn can also play a neuroprotective role by inhibiting α-syn aggregate and fiber formation (Kahle et al., 2000; Uversky et al., 2002; Sharma et al., 2020), V70M and P123H alter the structure and amyloidosis of β-syn, which accelerates the aggregate of β-syn (Ohtake et al., 2004). γ-syn is structurally similar to α-syn and can form fibers alone, but its formation rate is much slower than that of α-syn.

Interactions between members of the synaptic nucleoprotein family have been widely discovered, but whether β-syn, γ -syn is involved in the regulation of α-syn LLPS is currently unknown. Observing the effect of β-syn, γ-syn on α-syn condensate formation by LLPS may provide us with new insights to study the interactions between synaptic nucleoproteins and prevent abnormal aggregate of α-syn.

## 3. Phase separation in mitochondrial homeostasis in Parkinson’s disease

### 3.1. Overview of mitophagy

Cellular homeostasis is an important basis for the organism to maintain normal physiological activities. Therefore, maintaining cellular homeostasis is of great significance for disease prevention and treatment. Currently, cells maintain homeostasis by removing damaged components through two main pathways: (1) Ubiquitin-proteasome system (UPS): degrades short-lived proteins in cells. (2) Autophagy-lysosome pathway (ALP): digests long-lived proteins in cells and participates in the autophagy of abnormal organelles (Nijholt et al., 2011). These two systems are synergistically involved in maintaining the normal function of neurons, and when they become dysfunctional, they trigger the development of neurodegenerative diseases, such as PD.

Cellular autophagy plays an essential role in maintaining cellular metabolic homeostasis and involves a series of processes such as double membrane formation, extension, vesicle maturation (called autophagosomes), and translocation of target cargo to lysosomes (Glick et al., 2010; Ghavami et al., 2014). Mitochondria are important double-membrane organelles in the cell and play a role in fundamental processes of cellular activity such as ATP production, calcium signaling, and iron homeostasis (Raffaello et al., 2016; Spinelli and Haigis, 2018). Mitophagy is the process by which damaged and aged mitochondria are delivered to lysosomes for degradation via the autophagic pathway for mitochondrial quality and quantity regulation (Pickles et al., 2018; Onishi et al., 2021). Mitophagy is a crucial way for cells to prevent damaged mitochondria accumulation and perform mitochondrial quality control (Liu et al., 2019).

Narendra et al. (2008) first revealed PINK1-Parkin-mediated mitophagy and demonstrated that it is one of the key pathways of mitophagy (Nardin et al., 2016; Kumar et al., 2017). PINK1 is a mitochondrial targeting protein that enters mitochondria under physiological conditions via the translocase of the outer membrane (TOM) complex on the outer mitochondrial membrane (OMM) and the inner membrane translocase (TIM) 23 complex on the translocase of the inner membrane (IMM) (Pickrell and Youle, 2015; Sekine and Youle, 2018). When PD induced mitophagy occurs, mitochondria undergo depolarization, PINK1 accumulates on the OMM, and the S228 and S402 sites undergo autophosphorylation while phosphorylating the S62 site of the Parkin Ubl structural domain, recruiting and activating the E3 ubiquitin ligase Parkin, which in turn ubiquitinates mitochondrial outer membrane-associated proteins and recruits autophagy receptors (e.g., p62/SQSTM1, etc.), isolate and translocate damaged mitochondria for degradation via the autophagy-lysosome pathway, and mediate mitophagy (Narendra et al., 2008; Matsuda et al., 2010; Ziviani et al., 2010; Heo et al., 2015; Lazarou et al., 2015; Wauer et al., 2015; Han et al., 2020). Dysregulation of mitophagy may contribute to the neurodegenerative pathogenesis of PD.

Mitophagy plays an important role in PD. Abnormal mitochondrial function and damage are present in patients with PD, mitophagy can remove the damaged mitochondria and reduces mitochondrial dysfunction and mitochondria-associated cytotoxicity (Malpartida et al., 2021). This helps maintain the balance of energy metabolism within the cell and the healthy state of the mitochondria. Furthermore, mitochondria are one of the major intracellular sources of oxidative stress, and when mitochondrial function is impaired, it leads to excessive oxidative stress and mitochondrial DNA damage (Lizama and Chu, 2021). Mitophagy reduces oxidative stress and mitochondrial DNA damage, thereby protecting neurons from damage. Therefore, mitophagy is considered a potential therapeutic target for PD.

### 3.2. LLPS in mitophagy

**Figure 2:**
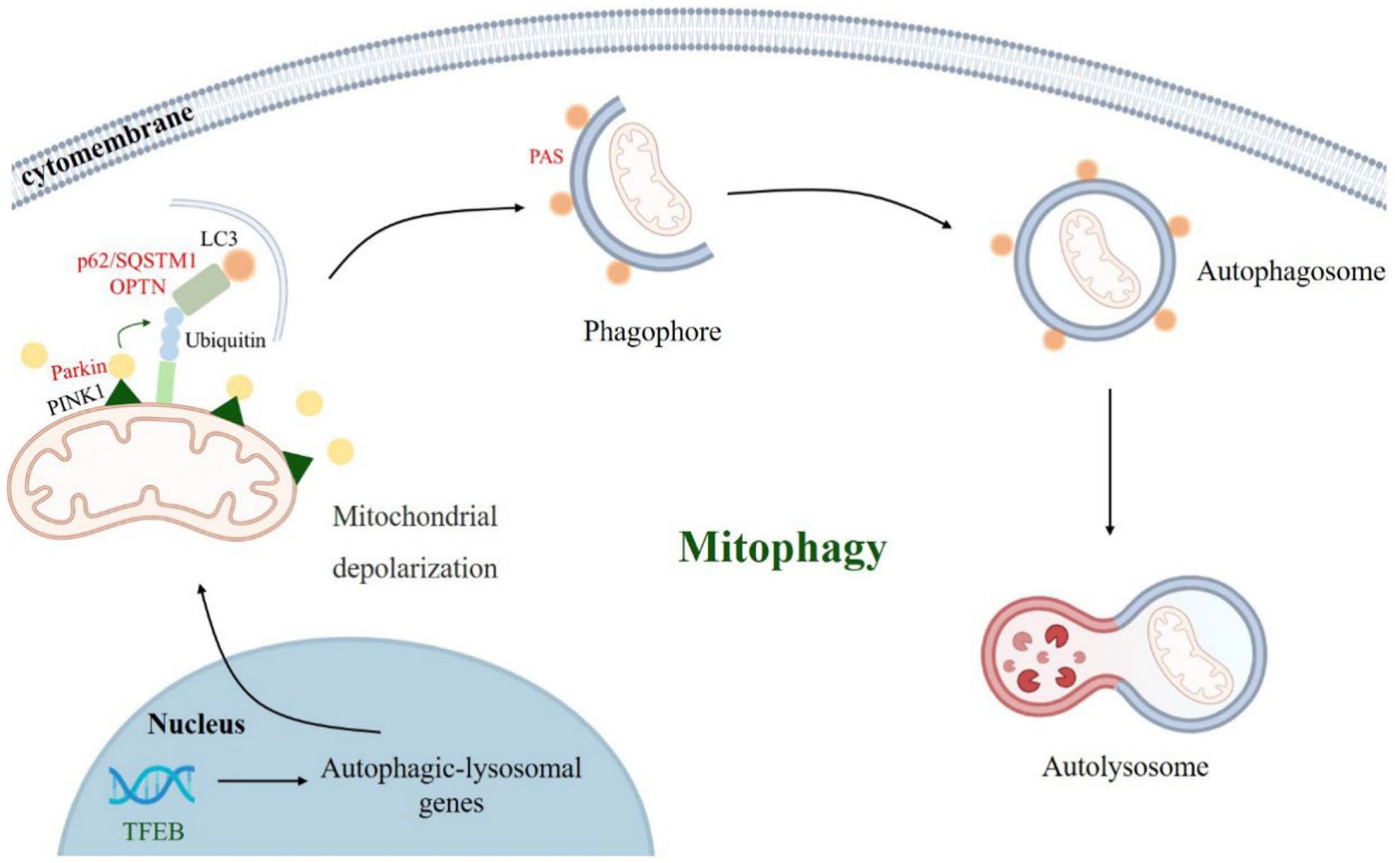
PINK1-Parkin-mediated mitophagy process. When Parkinson’s disease occurs, mitochondria depolarize, PINK1 accumulates in the mitochondrial outer membrane and recruits parkin. Parkin ubiquitinate mitochondrial outer membrane proteins and recruit autophagy receptors (such as p62/SQSTM1, OPTN, etc.), which bind to LC3 and other proteins to mediate autophagosome formation and autophagy-lysosomal pathway.

Recently, LLPS has also been observed in mitophagy (Hollenstein and Kraft, 2020; Noda et al., 2020; Darling and Shorter, 2021; Hayashi et al., 2021; Zhang, 2022), Parkin, autophagy receptor p62/SQSTM1, TFEB, and several other biomacromolecules play important roles in mitophagy (Figure 2). However, the mechanisms by which these biomolecules undergo selective autophagy, how their biophysical properties affect their physiological functions, and whether they are associated with susceptibility to autophagic degradation remain unclear. Here we highlight recent studies on phase separation in PINK-Parkin-mediated mitophagy and explore the role and mechanisms of biomolecular condensates in regulating PINK-Parkin-mediated selective autophagy (Table 1).

**Table 1:** Proteins that undergo LLPS during mitophagy in Parkinson’s disease.

Protein	Character	Key domain	Condition	Effect
Parkin	E3 ubiquitin ligase	IBR	Parkin is activated by PINK1 and interacts with a variety of E2 ubiquitin coupled enzymes, and LLPS occurs	Mediate ubiquitination of mitochondrial outer membrane substrate proteins
p62/SQSTM1	Autophagy receptor protein	PB1 UBA	LLPS occurs upon binding of p62/SQSTM1 to ubiquitinated substrate proteins	Mediate phagophore formation and eventually form autophagosomes
OPTN	Autophagy receptor protein	UBA	LLPS occurs when OPTN binds to ubiquitinated proteins	Mediate phagophore formation and eventually form autophagosomes
TFEB	transcription factor	bHLH	TFEB condensates are observed in Hela cells, but the conditions for its occurrence remain unclear	Activate autophagy-lysosome gene expression and participates in the autophagy-lysosome pathway
PAS	A variety of ATG proteins involved in the PAS	A variety of ATG proteins LCD domain	Atg13 and Atg17-Atg29-Atg31 complexes serve as scaffold proteins, and Atg1 is recruited to the condensate to function	Recruitment of ATG proteins for further autophagosome formation

#### 3.2.1. LLPS of Parkin

Parkin is an E3 ubiquitin ligase of the RING-between-RING (RBR) family consisting of 465 amino acids, including a ubiquitin-like (Ubl) structural domain (residues 1–76) and four zinc-ligated cyclic structural domains: RING0 (residues 141–225), RING1 (residues 226–327), IBR (residues 328–378) and RING2 (residues 410–465) in five parts (Koyano et al., 2014). The IBR structural domain is prion-like and is thought to play a critical role in Parkin phase separation to form condensates. By adding Azami-Green fusion tags to ubiquitin-coupled enzyme (E2) and Ash (Assembly helper) fusion tags to ubiquitin ligase (E3), Ryota et al. observed the interactions between them and found that during mitophagy, multiple E2s interact with activated Parkin to form a liquid–liquid phase separation within the cell, which subsequently triggers activation of E3 and subsequent ubiquitination of the substrate (Hayashida et al., 2023).

Previous studies have found that in PD pathology, PINK1 accumulation on the OMM triggers low-level ubiquitination and phosphorylation of Parkin, and activated Parkin ubiquitinates OMM proteins, providing more substrates for PINK1 and accelerating Parkin recruitment to the mitochondria and activation. PINK1 plays a key role in Parkin recruitment and activation, and it is known that Parkin undergoes LLPS to form liquid condensate under normal physiological conditions, so whether Parkin will still undergo LLPS and then be activated after PINK1 removal, and how its liquid condensate biophysical properties will be changed. This provides a new perspective further to investigate the role of PINK1/Parkin in mitophagy.

**Figure 3:**
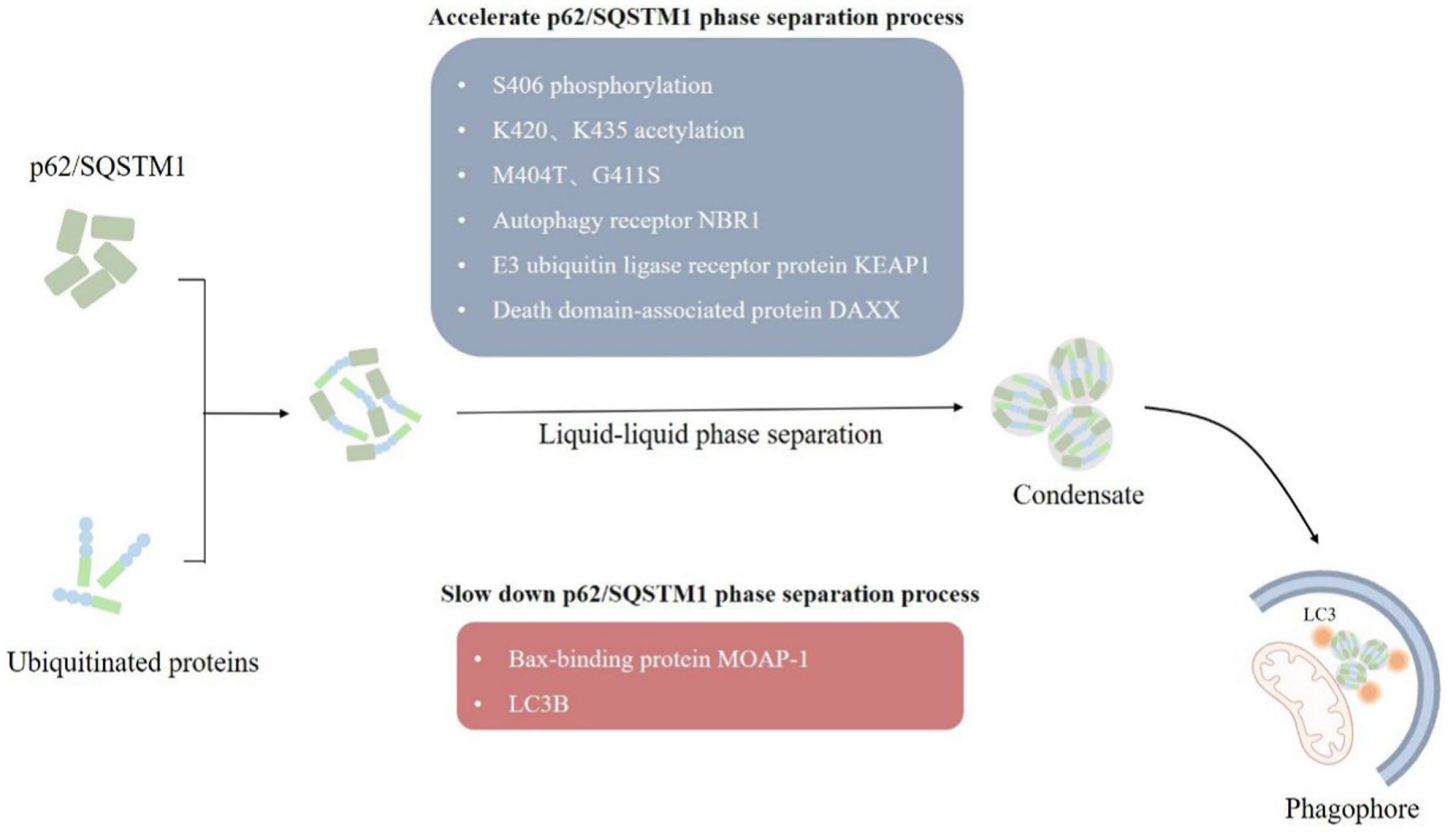
LLPS of p62/SQSTM1. (1) Autophagy receptors p62/SQSTM1 recognize and bind to ubiquitylated substrate proteins, form p62/SQSTM1 aggregates through the liquid–liquid phase separation mechanism, which in turn mediate the formation of phagophore, and finally degrade damaged mitochondria by forming autophagosomes. (2) A variety of factors, such as post-translational modifications, point mutations, and multiple interacting proteins influences the formation of p62/SQSTM1 condensate.

Post-translational modifications associated with the PINK1/Parkin pathway (e.g., phosphorylation, ubiquitination, etc.) play a key role in the regulation of mitophagy (Li et al., 2023). Different isoforms of phosphatase and tensin homologs (PTEN) have been found to be involved in the regulation of mitophagy. Subtype PTEN-a regulates mitophagy by promoting PINK1-mediated ubiquitin phosphorylation and accelerating the recruitment of Parkin in damaged mitochondria (Li et al., 2018; Barazzuol et al., 2020). The other isoform, PTEN-L, inhibits mitophagy by antagonizing ubiquitin phosphorylation, preventing Parkin mitochondrial translocation and inhibiting its E3 ubiquitin ligase activity (Wang et al., 2018). In addition, protein phosphatase with EF-hand structural domain 2 (PPEF2) was shown to inhibit PINK1-dependent mitophagy through ubiquitin dephosphorylation, acting as a negative regulator of mitophagy (Wang et al., 2018; Wall et al., 2019). Various deubiquitinases (DUBs) such as USP8, USP14, USP15, USP30, USP35, etc. negatively regulate mitophagy by interacting with parkin or its substrates to antagonize parkin activity (Eiyama and Okamoto, 2015; Harper et al., 2018). Could we also observe the effect of different post-translational modification effectors (e.g., PTEN isoforms, DUBs, etc.) on parkin by phase separation? This may provide a new starting point to further clarify its interaction mode.

#### 3.2.2. LLPS of p62/SQSTM1

p62/SQSTM1 mediates selective autophagy of ubiquitinated protein aggregates (Bjorkoy et al., 2005). p62/SQSTM1 consists of 440 amino acids with Phox and Bem1 (PB1) structural domains at the N terminus, followed by ZZ-type zinc finger motifs, LC3 interaction region (LIR), Keap1 interaction region (KIR), and ubiquitin-associated (UBA) structural domain at the C terminus (Ichimura et al., 2008; Sanchez-Martin et al., 2019). The PB1 and UBA structural domains of p62/SQSTM1 mainly mediated the onset of phase separation (Berkamp et al., 2021). We observed the formation of p62/SQSTM1 condensates *in vivo*, and phase separation occurred when reconstituted p62/SQSTM1 mixed with polyubiquitin chains *in vitro*, and the cohesive bodies exhibited a semi-liquid nature (Figure 3). In addition, the phase separation process of p62/SQSTM1 is influenced by several factors, such as post-translational modifications of the protein like phosphorylation, acetylation (You et al., 2019; Fujioka and Noda, 2021), p62/SQSTM1 disease-associated mutations (M404T, G411S) (Sun et al., 2018; Zaffagnini et al., 2018), interacting proteins (NBR1, Nur77, KEAP1, MOAP-1, DAXX, etc.) (Yang et al., 2019; Sanchez-Martin et al., 2020; Peng S. Z. et al., 2021). Phosphorylation of S406, acetylation of K420 and K435, M404T and G411S mutations, autophagy receptor NBR1, E3 ubiquitin ligase receptor protein KEAP1, and death domain-associated protein DAXX all promote p62/SQSTM1 phase separation and increase condensate mobility and autophagic degradation (Lee et al., 2017; Yang et al., 2019; You et al., 2019; Sanchez-Martin et al., 2020). The N-terminal IDR domain of Nur77 interacts with the N-terminal PB1 domain of p62/SQSTM1 to form a Nur77-p62 condensate, isolating damaged mitochondria and transferring them to lysosomes (Berkamp et al., 2021; Komatsu, 2022). In contrast, the Bax-binding protein MOAP-1 interacts with the PB1-ZZ structural domain of p62, interfering with the self-oligomerization and liquid–liquid phase separation process of p62 (Tan et al., 2021). LC3B was also found to regulate p62/SQSTM1 phase separation negatively.

p62 acts as an autophagy receptor that recognizes depolarized mitochondria through the ubiquitin chain and isolates and transfers damaged mitochondria by interacting with LC3. p62 has also been observed to interact with the upstream factor of the isolation membrane, FIP200, to recruit the ULK1 protein kinase complex and autophagy-related (ATG) protein to form the isolation membrane (Fan et al., 2010; Jain et al., 2010; Danieli and Martens, 2018). Later, we may be able to further clarify the role of p62 in mitophagy by observing exactly how PINK1-Parkin-mediated ubiquitination of OMM proteins is involved in the altered nature of p62 condensate and whether phase transitions occur during the interaction of p62 with LC3 and ATG proteins.

#### 3.2.3. LLPS of OPTN

OPTN acts as an autophagy receptor protein that interacts with LC3 to connect damaged mitochondria to autophagosomes. Phase separation of OPTN has also been observed. Yamano et al. observed the occurrence of OPTN LLPS by using Fluoppi’s protein–protein interaction technique, which fused Ash tags to ubiquitin chains and humanized AzamiGreen (hAG) tags to the autophagy receptor protein OPTN. Phase-separated condensates were formed through multivalent interactions between the ubiquitin chain and the ubiquitin-binding domain of OPTN. In addition, ATG9A has been observed to co-localize with the OPTN condensates and interact with OPTN in the process of phagophore and autophagosome formation. When this interaction is absent, mitophagy does not occur (Yamano et al., 2020).

Further observation of the altered biophysical properties of OPTN in mitophagy using phase separation and whether it interacts with key autophagy proteins such as Parkin and LC3 may help us to understand its biological functions in mitophagy further.

#### 3.2.4. LLPS of TFEB

Transcription factor EB (TFEB) is a key transcription factor that regulates the autophagy-lysosome system. It promotes the formation and function of autophagosomes and regulates mitophagy by promoting the transcription of autophagy-lysosome-related genes (e.g., PINK1, Parkin, LC3, etc.). TFEB undergoes LLPS to form condensate *in vitro* and in living cells, and IPMK which encodes inositol polyphosphate multi-kinase inhibits LLPS of TFEB. *In vitro* purified maltose-binding protein (MBP)-tagged TFEB undergoes LLPS in the presence of 200 μM NaCl and 5% PEG-8000, and the addition of IMPK was found to inhibit the formation of TFEB droplets (Chen et al., 2020). Phase separation of TFEB has also been observed in Hela cells. When IPMK is absent, the number of TFEB condensates and their co-localization with MED1 and target mRNAs increases, and activates TFEB, which in turn promotes autophagy and lysosomal biogenesis (Chen et al., 2020; Ferrari and Martens, 2020). Biological small molecules such as Ro-3,306 increase TFEB condensate body size and propensity to fuse, promoting lysosomal biogenesis and function in a TFEB-dependent manner and autophagy (Wang et al., 2022).

TFEB activates genes such as PINK1 and LC3 and promotes the formation of autophagic vesicles for degradation. In addition, TFEB can also inhibit the expression of genes such as mfn1 and mfn2, which inhibit mitochondrial proliferation by suppressing the mitochondrial fusion process. Whether these autophagy gene-expressed proteins can regulate TFEB biological activity by altering the biophysical properties of TFEB condensate bodies may be a starting point for us to explore other functions of TFEB.

#### 3.2.5. LLPS of PAS

The formation of preautophagosomal structures (PAS) in the early stages of autophagy is closely related to LLPS, and Fujioka et al. found that the interaction between ATG1 complexes containing IDR structural domains resulted in phase separation *in vitro* to form a liquid condensate, which is PAS. Also, the mobility of PAS is essential for its dynamic recruitment of ATG proteins during autophagosome formation. The mobility of PAS is crucial for its dynamic recruitment of ATG proteins during autophagosome formation. For example, it activates ATG1 kinase to initiate autophagy and facilitates the binding of ATG9 vesicles to promote the formation of synaptic autophagosomal membrane vesicles. PAS formation is also inhibited when ATG1 undergoes point mutations or phosphorylation inhibition phase separation occurs (Fujioka et al., 2020).

As the initial step of autophagy, the PAS is the basis for the subsequent formation of complete autophagosomes. The unique fluidity of the liquid condensate formed by LLPS may be ideal for forming autophagosome assemblies, and LLPS may continue to play an important role in subsequent autophagosome formation.

Mitophagy is a key process in Parkinson’s disease and many important human neurodegenerative diseases. As an important mechanism regulating the process of mitophagy, understanding the changes of autophagy-related protein LLPS provides new ideas for the study of mitophagy and new solutions for the pathological study, diagnosis, and treatment of Parkinson’s disease and many mitochondria-related diseases.

## 4. Summary

There is currently no effective treatment for PD, so an in-depth understanding of its pathogenesis is of great significance for treating of PD. There is increasing evidence that liquid–liquid phase separation is involved in the pathological process of PD, that phase separation to form biomolecular condensates is involved in abnormal α-syn aggregate and mitophagy, and that altered biophysical properties of condensates are involved in regulating protein aggregate and mitophagy processes in PD.

LLPS of α-syn occurs at the pre-nucleation stage of its aggregate, and delaying the process of α-syn LLPS may serve as a new target for PD treatment. Mitophagy plays a crucial role in the clearance of damaged mitochondria in PD, and various key mitophagy proteins and related structures have been found to undergo LLPS. In addition, there is also a close relationship between α-syn aggregate and mitophagy, and α-syn aggregate negatively affects the regulation of mitophagy. Further studies exploring the relationship between α-syn and mitophagy will help unravel PD’s pathogenesis and provide new ideas for developing relevant therapeutic strategies. However, it is unclear whether LLPS is involved in the interaction process between α-syn and mitophagy. Therefore, to further understand PD pathology, it is essential to reveal the interaction of phase separation with abnormal α-syn aggregate and mitophagy.

## Author contributions

KH collected the data and material and wrote the manuscript. TL, JL, and MX helped study, collect and analyze the data. LS and JW helped conceive the study and revise the manuscript. All authors have read, discussed, and approved the manuscript.

## Funding

This work was supported partly by the National Natural Science Foundation of China (32161143021, 22203027, and 81271410), and Henan Natural Science Foundation of China (182300410313).

## Conflict of interest

The authors declare that the research was conducted in the absence of any commercial or financial relationships that could be construed as a potential conflict of interest.

## Publisher’s note

All claims expressed in this article are solely those of the authors and do not necessarily represent those of their affiliated organizations, or those of the publisher, the editors and the reviewers. Any product that may be evaluated in this article, or claim that may be made by its manufacturer, is not guaranteed or endorsed by the publisher.
